# Successful treatment of a patient with renal failure, treated with haemodialysis, and advanced ovarian germ cell tumour using modified cisplatin-based chemotherapy duplet

**DOI:** 10.3332/ecancer.2022.1397

**Published:** 2022-05-23

**Authors:** Viridiana Méndez-Calderillo, Gerardo Nuñez-Saldaña

**Affiliations:** Women’s Cancer Clinic, Hospital Materno Celaya, Celaya, Guanajuato 38065, Mexico

**Keywords:** haemodialysis, ovarian germ cell cancer, chemotherapy, kidney failure, cisplatin

## Abstract

There are no reports on chemotherapy treatment in patients with ovarian germ cell tumours and kidney failure. We report the case of a 29-year-old female diagnosed with an advanced right ovarian germ cell tumour and severe kidney damage treated with haemodialysis. The first cycle of chemotherapy was administered with 10 mg/m2 of cisplatin on days 1, 3, and 5, and 35 mg/m2 of etoposide from day 1 through 5, followed by haemodialysis 1 hour after the end of cisplatin infusion on days 1, 3, and 5, with grade 3 haematologic toxicity. After the first cycle, kidney function improved and haemodialysis was suspended. From the second cycle onwards, the dose was increased to 80% cisplatin and 100% etoposide with grade 3 haematologic toxicity; following that, the dose of etoposide was decreased to 80% in cycle 3. In the face of tumour progression, the regimen was changed to a standard dose of ifosfamide on days 1 through 5, and carboplatin on day 1 calculated at the area under the plasma concentration curve of free carboplatin versus time of 5 mg/ml/minute. An 80% dosage of ifosfamide and cisplatin was used from the second to fourth cycle, achieving partial response by imaging. The patient was taken to surgery and there was no histopathological evidence of viable cancer cells. In conclusion, cisplatin-based chemotherapy can be administered to a patient with advanced ovarian germ cell tumour and renal insufficiency at lower doses to prevent side effects while retaining efficacy in a multidisciplinary treatment setting.

## Introduction

Ovarian germ cell tumours are malignant neoplasms that represent about 5% of all ovarian tumours and typically appear in young women, predominantly around the age of 20 [1]. Because of the rarity of these tumours, their treatment has been based on randomised controlled clinical trials of testicular germ cell tumours [2].

Ovarian germ cell tumours have a very high cure rate since the introduction of cisplatin. With multimodality management, 5-year survival exceeds 85%. Surgical resection represents the first step in the management of these tumours. However, even after complete resection, the risk of recurrence of the tumour is in the 25%–100% range, depending on cell type and stage; so post-operative cisplatin-based chemotherapy is the treatment of choice. Patients with advanced disease who are not candidates for initial surgery are offered preoperative chemotherapy. This approach has not been tested in clinical trials because most ovarian germ cell cancers are diagnosed and treated in early stages. Preoperative chemotherapy regimens do not differ from those used post-operatively. All are based on platinum, and the 5-day bleomycin, etoposide and cisplatin (BEP) regimen is the most commonly used. Currently, the recommendation is three cycles in the case of early tumours and four cycles for advanced stages. The BEP regimen consists of 20 mg/m^2^ of cisplatin and 100 mg/m^2^ of etoposide on days 1 through 5, and 30 units weekly of bleomycin at 21-day intervals. The EP regimen (etoposide and cisplatin) can be used in the case of patients who are ineligible for bleomycin, such as those advanced in age, with poor kidney function, history of tobacco use or pulmonary comorbidities, due to the risk of pneumonitis induced by bleomycin [2, 3].

Between 70% and 80% of the patients with advanced disease experience a complete response to initial treatment of chemotherapy with cisplatin and etoposide, with or without bleomycin. The remaining patients will develop refractory disease with few potentially curative chemotherapy treatment options. The management of these patients is complex due to the scarcity of data and is primarily based on information extrapolated from the male population and reports of institutional experiences. The paclitaxel, ifosfamide and cisplatin (TIP) regimen consisting of 250 mg/m^2^ of paclitaxel on day 1, 1,500 mg/m^2^ of ifosfamide per day from day 2 through 5 and 25 mg/m^2^ per day of cisplatin from day 2 through 5 is commonly used in this scenario [3, 4].

Given that 90% of cisplatin is eliminated via the kidneys, several manufacturers and clinical guides recommend abstaining from cisplatin treatment in patients with a glomerular filtration rate below 60 ml/minute. Because of this, patients with pre-existing kidney failure usually receive carboplatin, which is less toxic than cisplatin but also less effective [5].

The administration has been attempted in scenarios of moderate to severe kidney failure, even in patients with haemodialysis, in highly curable tumours with cisplatin-based regimens. Some authors have reported using cisplatin at 50% of the dose for haemodialysis patients in the successful treatment of some kinds of cancer, such as lung, ovarian and testicular cancer, but with increased haematologic toxicity due to the accumulation of drug metabolites [6–8].

This is the first reported case describing the use of cisplatin concomitant with haemodialysis in a patient with ovarian germ cell cancer.

## Clinical case

**Figure 1.**
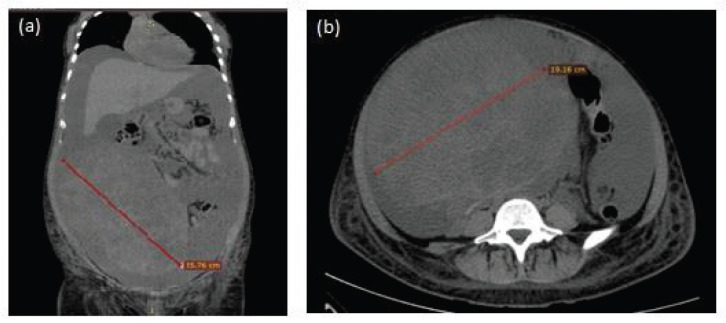
Representative images of simple abdominal computerised tomography (CT) scan. (a) Coronal section showing 25.76 cm (red line) heterogeneous abdominopelvic tumour and abundant ascites fluid. (b) Axial section with 19.16 cm tumour (red line).

A 29-year-old woman presented with a history of caesarean and bilateral tubal occlusion in November of 2018. She was referred to the oncology department in February of 2019, with diagnosis of a right ovarian germ cell tumour IVa (Figure 1) with alpha-fetoprotein (2.19 ng/mL; normal: <6.1 ng/mL), lactate dehydrogenase (10,090 U/L; normal: 240–480 U/L), ß-hCG fraction (279.10 mUI/mL; normal: <5 mUI/mL), creatinine (9.22 mg/dL; normal: 0.5–1.3 mg/dL) and urea (181 mg/dL; normal: 15–38 mg/dL). Upon hospital admission, she required management by the intensive care unit due to septic shock secondary to urinary infection from *Escherichia coli*. She presented with respiratory failure that required intubation and anuria that was treated with haemodialysis. She completed carbapenem antibiotic treatment and was discharged from the intensive care unit still experiencing kidney failure, with haemodialysis replacement treatment every third day.

The patient was evaluated by the functional oncology unit, concluding that she was not a candidate for initial surgery due to advanced tumour disease. In order to provide the best chance for cure, preoperative chemotherapy was proposed with a cisplatin and etoposide chemotherapy regimen at an adjusted dosage, omitting bleomycin because of the potential pulmonary toxicity, given her recent advanced airway management. Mention was made of the non-standard use of cisplatin in patients with kidney failure, the scientific evidence supporting its application and the analysis of potential risks and benefits of treatment respecting the principles of non-maleficence, beneficence, autonomy and justice. Ultimately, the patient authorised the treatment by signing the informed consent.

The patient received haemodialysis three times a week; thereafter, the regimen proposed by Kamizuru *et al* [9] was decided upon. The first cycle of chemotherapy was received at the hospital via central venous catheter and consisted of 50% of the standard dosage of cisplatin (10 mg/m^2^) on days 1, 3 and 5, and 35% of the standard dose of etoposide (35 mg/m^2^) from day 1 through 5. The haemodialysis was carried out an hour after the end of the cisplatin infusion. The patient developed grade 2 mucositis by day 6+ and granulocyte colony-stimulating factor was applied at 5 μg/kg per day from day 7 through 11. Additionally, she presented with grade 3 anaemia which was managed with blood transfusions.

After the first cycle, there was improvement in kidney function with the suspension of haemodialysis and the tumour markers returned to normal. The second cycle was delayed by 14 days because of the isolation of *Staphylococcus aureus* in the central venous catheter and the patient was hospitalised in order to remove the central venous catheter and for treatment with ceftazidime.

The second chemotherapy cycle was indicated with a standard dose of etoposide (100 mg/m^2^ on day 1 through 5) and cisplatin at 80% of the standard dosage (16 mg/m^2^ day 1 through 5) on a glomerular filtration rate calculated with the Cockcroft–Gault formula at 53 mL/minute and was accompanied by a granulocyte colony-stimulating factor for 5 days. The patient again developed grade 3 anaemia which was managed with blood transfusions. In the third chemotherapy cycle, the etoposide dose (80 mg/m^2^ day 1 through 5) was decreased by 20% and the cisplatin dosage remained unchanged presenting as grade 2 anaemia toxicity.

After the third cycle, the patient developed lumbar pain, weight loss, abdominal tumour growth and increased creatinine levels. With a glomerular filtration rate of 31 mL/minute, a second line of chemotherapy with a full dosage of ifosfamide 1,200 mg/m^2^/day on days 1 through 5 was begun, and carboplatin on day 1 was calculated as the area below the free carboplatin plasma concentration versus time curve of 5 mg/ml/minute with colony-stimulating factor at 5 kg/kg/day for 6 days. Paclitaxel was omitted because of clinical deterioration and a history of haematologic toxicity with chemotherapy duplet. The patient again presented with grade 3 anaemia and also a tumour response with clinical and kidney function improvement with a glomerular filtration rate calculated at 52 ml/minute where the second to fourth cycle changed the carboplatin for cisplatin to 80% of the standard dosage (16 mg/m^2^/day on days 1 through 5) and ifosfamide at 80% of the standard dosage (1,000 mg/m^2^/day on days 1 through 5), presenting as the only haematologic toxicity grade 2 anaemia.

**Figure 2.**
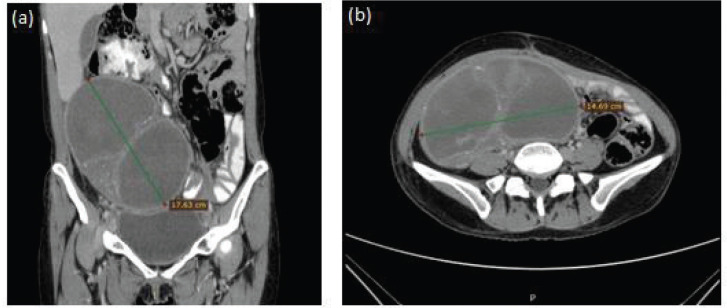
Representative images of contrast-enhanced abdominal CT scan showing partial tumour response. (a) Coronal section with 17.63 cm tumour (green line) and without ascites. (b) Axial section with 14.69 cm tumour (green line).

A CT scan was done with abdominal contrast showing partial tumour response (Figure 2) and an optimal cytoreduction was performed on 5 December 2019 with a histopathological report of necrosis and no evidence of residual tumour in the material analysed.

The patient has 28 months of disease-free survival and functional status of 0 on the Eastern Cooperative Oncology Group scale.

## Discussion

Standard chemotherapy for the treatment of germ cell ovarian cancer is based on cisplatin [3]. However, patients with kidney failure were not included in the clinical trials supporting this indication. These patients represent a challenge for medical oncologists since the decreased kidney excretion favours the accumulation of drugs, causing greater toxicity, and chemotherapy in this clinical setting has never been well established [5, 6]. The CANDY study conducted in France, on the clinical practice of chemotherapy in cancer patients with kidney failure treated with haemodialysis, reports that 44% of the patients developed iatrogenic toxicity because of an inappropriate dose adjustment due to the lack of recommendations for the management of cytotoxins in this group of patients [10].

Because chemotherapy for germ cell tumours should be given with a curative purpose, some reports suggest that in patients with decreased kidney function a reduced dose of cisplatin can be administered because 90% of a drug is eliminated via the renal tract. However, the doses recommended differ among authors and such modification of dosage can carry a risk of undertreatment [5].

Few reports of full remission exist in spite of an adjustment of cisplatin dosage in the treatment of advanced testicular cancer in patients with kidney and renal replacement therapy [7, 9].

Cisplatin nephrotoxicity is no longer a limitation in haemodialysis patients, but this is not the case for other dose-dependent toxicities such as anaemia and neuropathy [5]. It would be better to reduce the cisplatin dosage to approximately 50% and perform hemodialysis immediately after the administration of cisplatin given that the pharmacological effects of platinum derivatives can not only be attributed to the short-term effects of drug diffusion in tissues, but also to the more delayed effects of circulating platinum strains. In the case of etoposide, its cytotoxicity depends on the concentration as well as on the duration of exposure; with a urinary output of 40.8% of the dosage, its half-life elimination will increase significantly in the case of kidney failure. A reduction in the dosage to 60% in haemodialysis patients is recommended given its high volume of distribution and very long plasma half-life [11].

In this patient’s case, the first chemotherapy cycle with cisplatin and etoposide was calculated following the dose adjustment which was recommended by Kamizuru *et al* [9]; haemodialysis began an hour after chemotherapy infusion in order to decrease the haematologic toxicity [6, 8] and with improvement in kidney function, the duplet dose was escalated. With respect to toxicity, anaemia was most important since the patient required support with multiple erythrocyte concentrates to be able to continue treatment cycles. Additionally, chemotherapy was delayed due to bacterial infection, a situation that has been previously reported in this scenario [7].

## Conclusion

As far as we know, this is the first successful chemotherapy description in a patient with kidney failure and advanced ovarian germ cell tumour. In the literature, there are few references regarding the adjustment of chemotherapy doses in patients with renal dysfunction. For this reason, it is common for complications associated with an overdose or, on the contrary, little response in tumour remission to be present. This case suggests that chemotherapy treatment, with mainly cisplatin dose adjustment, is possible for haemodialysis patients with a favourable outcome. However, secondary toxicity risk should be taken into account for the administration of chemotherapeutic agents, which require close clinical follow-up, as well as the early management of their possible complications. Therefore, the participation of a multidisciplinary team of doctors specialising in oncology, nephrological and intensive therapy is recommended in order to optimise the administration and logistics of the treatment of these patients.

## Patient’s perspective

‘We are very grateful to all the doctors who cared for me at the Celaya Maternity Hospital, thanks to them and to God I am still alive and I can take care of my children. Since my recovery, each time I meet someone with cancer, I recommend that it is treated quickly so that it can be cured’.

## Informed consent

Informed patient consent was obtained prior to the case report.

## Conflicts of interest

The authors declare that they do not have any conflicts of interest.

## Funding

The authors did not receive funding for the preparation of this manuscript.

## Authors’ contributions

All authors participated in the bibliography research and preparation of the manuscript. All authors approved the final version of the same.
